# M2-Macrophage-Derived Exosomes Promote Meningioma Progression through TGF-*β* Signaling Pathway

**DOI:** 10.1155/2022/8326591

**Published:** 2022-05-20

**Authors:** Xiao-Hong Fu, Jian-Ping Li, Xue-Ying Li, Yan Tan, Min Zhao, Shao-Fu Zhang, Xue-Dong Wu, Jian-Guo Xu

**Affiliations:** ^1^Department of Neurosurgery, West China Hospital, Sichuan University, Chengdu 610041, China; ^2^Department of Neurosurgery, The First People's Hospital of Zunyi, Zunyi, 563000, China; ^3^Department of Ophthalmology, Affiliated Hospital of Zunyi Medical University, Zunyi, Guiyang, China; ^4^Department of Pharmacy, The First People's Hospital of Zunyi, Zunyi, 563000, China

## Abstract

Tumor-associated macrophages (TAMs) have been shown to be an essential component of the tumor microenvironment and facilitate the proliferation and invasion of a variety of malignancies. However, the contribution of TAMs to meningioma progression has not been characterized in detail. In this study, we aimed to discover a novel regulatory pathway by which exosome-mediated M2-polarized macrophages participate in meningioma tumorigenesis and progression. *Methods*. First, the distribution and functional phenotype of macrophages in meningioma tissues were assessed by immunohistochemistry. Macrophage-derived exosomes (MDEs) were characterized, and further cell coculture experiments were performed to explore the effects of M2-MDEs on the proliferation, migration, and invasion of meningioma cells. RNA sequencing was used to analyze the transcriptomic signatures in meningioma cells treated with M2-MDEs. Three-dimensional tumorspheres and xenograft tumor models were used to evaluate the effects of M2-MDEs on meningioma tumorigenesis and development. *Results*. We found that M2 macrophages were enriched in meningioma tissue. Coculture with meningioma cells induced the M2 polarization of macrophages. We also found that M2-MDEs were able to significantly promote cell proliferation, cell migration, cell invasion, and tumorigenesis in meningiomas. Bioinformatic analysis suggested that the TGF-*β* pathway was activated in meningioma cells treated with M2-MDEs. Functional experiments demonstrated that blocking the TGF-*β* signaling pathway could effectively reverse the tumor-promotive effects mediated by M2-MDEs. *Conclusions*. Overall, our study showed that M2-MDEs promoted meningioma development and invasion by activating the TGF-*β* signaling pathway. Targeting exosome-mediated intercellular communication in the tumor microenvironment may be a novel therapeutic strategy for meningioma patients.

## 1. Introduction

As one of the most common primary tumors of the central nervous system, meningiomas arise from the arachnoid tissue surrounding the brain [1, 2]. Meningiomas can be defined as grade I (benign meningioma), grade II (atypical meningioma), and grade III (malignant meningioma) based on the grading criteria of the World Health Organization (WHO) [3]. The histopathological features of most meningiomas are benign (grade I), and patients with these meningiomas usually have an ideal prognosis with surgery [4]. Atypical and anaplastic meningiomas often exhibit biological characteristics of malignant tumors such as abnormal proliferative activity and aggressive growth patterns, which pose challenges for the treatment of meningiomas [5–7]. However, the molecular mechanisms of meningioma tumorigenesis, especially the role of the tumor microenvironment in its progression, are still underexplored.

Infiltrating macrophages are a critical immune cell population in a variety of tumor tissues, such as osteosarcoma, colorectal cancer, and lung cancer [8–10]. Influenced by the tumor microenvironment (TME), macrophages resident in tumor tissues undergo a transition of functional states and are also known as tumor-associated macrophages (TAMs). TAMs can be induced to polarize into M1 and M2 TAMs. Unlike M1 TAMs, which secrete proinflammatory factors (TNF*α*, IL-6, and IL-12), M2 TAMs are known to be anti-inflammatory-activated macrophages and can contribute to angiogenesis and tumor growth by regulating intercellular communication in the TME [11–13]. Meningioma is not restricted by the blood-brain barrier, which allows it to come into contact with peripheral immune cells more frequently [14, 15]. Several studies have shown that TAMs are enriched in meningioma tissue as a major immune cell population [16–18]. Although the functional status of infiltrating TAMs in meningioma tissues is closely related to the prognosis of patients, little is known about the potential role and the molecular mechanisms underlying the tumor-promoting effects of TAMs in meningioma proliferation and invasion [19, 20].

In recent years, exosomes have attracted much attention as mediators of intercellular communication in the TME [21, 22]. Mounting basic studies have reported that stromal-cell-derived exosomes play a critical role in regulating tumorigenesis and TME remodeling by transferring diverse contents such as miRNA, DNA, and cytokines [23–25]. Importantly, exosomes have been shown to be an important means through which macrophages interact with tumor cells. Zhang et al. demonstrated that macrophages promote colon cancer proliferation and invasion by targeting the PI3K/AKT and NF-*κ*B pathways via the exosome delivery of miR-183-5p [26]. Zheng and colleagues found that apolipoprotein E was specifically enriched in TAM-secreted exosomes and promoted the invasive ability of gastric cancer [27]. Nonetheless, it remains unknown whether macrophage-derived exosomes (MDEs) are involved in meningioma proliferation and invasion.

In this study, we examined the distribution of TAMs in meningioma tissues and constructed M2-polarized macrophages induced by cotreatment with IL-4 and IL-13. We demonstrated that M2-macrophage-derived exosomes (M2-MDEs) were able to effectively promote cellular viability, invasion, and tumor formation in malignant meningioma cells. RNA sequencing revealed activation of the TGF-*β* pathway in meningioma cells cocultured with M2-MDEs. Blockade of the TGF-*β* pathway can reverse the tumor-promoting biological effects of M2-MDEs. Our study not only reveals a novel mechanism of intercellular communication between meningioma cells and TAMs but also provides a promising molecular therapeutic target for precision medicine in meningioma.

## 2. Materials and Methods

### 2.1. Cell Culture and Treatment

Human meningioma cell lines (IOMM-Lee and CH157-MN) and human mononuclear macrophages THP-1 were bought from ATCC. IOMM-Lee and CH157-MN cells were passaged in DMEM medium supplemented with 10% exosome-free fetal bovine serum (FBS). THP-1 cells were cultured in RPMI 1640 medium supplemented with 10% exosome-free FBS. The medium was changed every 48 hours. THP-1 cells were induced to differentiate into M0 macrophages by treatment with 100 ng/mL of phorbol myristic acetate (PMA, Sigma) for 24 hours. To induce the M2 polarization of macrophages, 20 ng/mL IL-4 and 20 ng/mL of IL-13 were used to treat M0 macrophages for 48 hours. Transwell chambers (0.4 *μ*m, Corning) were used for coculturing meningioma cells (lower chamber) and macrophages (upper chamber). For the culture of 3D tumorspheres, 1000 meningioma cells were cultured in U-shaped microplates (Thermo Scientific) with a low-adhesion surface for 7 days.

### 2.2. Immunohistochemistry (IHC) and Immunofluorescence (IF) Staining

The study was approved by the Ethics Committee of the West China Hospital of Sichuan University. All the meningioma samples were obtained from West China Hospital. Paraffin sections were deparaffinized and rehydrated for antigen retrieval according to standard IHC and IF protocols [28]. Then, 3% H_2_O_2_ was used to block endogenous peroxidase, followed by the blocking of tissue sections. After overnight incubation with the primary antibodies at 4°C, the tissue sections were counterstained with hematoxylin after DAB staining. For IF staining, Alexa Fluor® 555-conjugated secondary antibody was used to incubate sections, and the nuclear chromatin is marked by DAPI staining. Sections were observed under an upright microscope, and image data were collected. The specific primary antibody information is listed in Supplementary Information Table S1.

### 2.3. RNA Extraction and Quantitative Real-Time PCR (qPCR)

Nucleic acids in the samples were extracted using TRIzol Reagent (Invitrogen, Waltham, MA, USA) according to the manufacturer's instructions. Briefly, dishes with 1 × 10^6^ cells were washed three times with PBS. 1 mL of TRIzol Reagent was used to lyse cells and release RNA. High-speed centrifugation was performed after mixing TRIzol with ethanol, and the mRNA was distributed in the upper aqueous phase. Collect mRNA using an adsorption column and dissolve it in 50 *μ*l RNase-free water. The 260/280 absorbance ratio (A260/A280) was used to assess RNA purity. A PrimeScript™ RT reagent Kit (TAKARA, Kusatsu, Japan) was used for the reverse transcription of mRNA. qPCR experiments were performed using the TB Green® Premix Ex Taq™ II kit (TAKARA). The amplification program was as follows: 95°C for 1 min, 40 cycles of 95°C for 5 s, and 60°C for 20 s. ACTB was used as the housekeeping gene to evaluate the relative expression level of the target gene. The PCR primers are listed in Supplementary Information Table S2.

### 2.4. Western Blot

Total protein from cells and tissues was extracted using RIPA lysis buffer (Beyotime). Cytoplasmic and nuclear proteins were extracted by sequentially disrupting the cell membrane and nuclear membrane through an osmotic pressure gradient using the Nuclear/Cytoplasmic Protein Extraction Kit (Beyotime). The protein extraction solutions were quantified using the BCA kit (Solarbio) and then thermally denatured using a metal bath. Proteins of different mass sizes were separated by vertical SDS-PAGE and then transferred to PVDF membranes. The PVDF membranes were incubated in blocking solution for 1 hour and then incubated with primary antibodies, followed by chemiluminescent detection using the ECL method. The specific primary antibody information is listed in Supplementary Information Table S1.

### 2.5. Exosome Isolation and Analysis

The exosomes secreted by macrophages were isolated using gradient centrifugation [29]. Briefly, macrophages were cultured for 48 hours in exosome-free medium. Then, the macrophage culture medium was collected and centrifuged at 1000 × *g* for 10 min, 3000 × *g* for 30 min, 6000 × *g* for 40 min, and 12,000 × *g* for 2 hours to remove the cell debris. The supernatant was collected and centrifuged at 100,000 × *g* for 4 h at 4°C to collect the exosomes in the pellet. The size and concentration of the exosomes were determined using transmission electron microscopy and a nanoparticle tracking analyzer. Evaluate the purity of exosomes by detecting the expression levels of exosome markers such as CD81, TSG101, CD9, and calnexin using western blotting. The exosomes were labeled with a PKH67 kit (Sigma, St. Louis, MO, USA) to detect the phagocytosis of exosomes by tumor cells.

### 2.6. Wounding Healing Assay

Meningioma cells (IOMM-Lee, CH157-MN) in the logarithmic growth phase were seeded in culture plates. When the cells reached 90% confluence, a scratch was made using a 200-microliter sterile pipette tip. Wash three times with PBS (without Ca^2+^ and Mg^2+^), then add medium containing 1% FBS, and take pictures under the microscope to record the original width of the scratches. The width of the scratch was recorded again after 24 h, and the migratory capacity of the tumor cells was assessed based on the distance of cell migration.

### 2.7. Cell Migration and Invasion Assay

Transwell assay (8.0 *μ*m) was used to evaluate the migration and invasion ability of meningioma cells with or without preincubated Matrigel (200 mg/mL, Corning). Briefly, meningioma cells (IOMM-Lee and CH157-MN) were added to FBS-free medium in the upper chamber, while medium containing 10% exosome-free FBS was placed in the lower chamber. After 24 h of incubation, cells that had passed through the membrane were fixed using 4% paraformaldehyde and stained with 0.1% crystal violet. Images under random fields of view were collected under an inverted microscope, and the number of tumor cells penetrating the membrane was counted.

### 2.8. Cytokine Measurements

The secretion of IL-10 by macrophages was quantified using an ELISA kit (R&D Systems, Minneapolis, MN, USA). Media from different groups of macrophages were collected for testing, after centrifugation to remove cellular debris, according to the manufacturer's instructions.

### 2.9. Apoptosis Assays

Apoptotic cells among the tumor cells were detected using a FITC-labeled Annexin V Apoptosis Detection Kit (Biolegend) according to the manufacturer's instructions. The ratio of apoptotic cells was detected by flow cytometry.

### 2.10. RNA Sequencing and Raw Data Preprocessing

TRIzol Reagent (Invitrogen) was used to lyse the meningioma cell line IOMM-Lee for extraction and purification of total RNA. The RNA quality was determined by examining the A260/A280 with a NanoDrop™ OneC spectrophotometer (Thermo Fisher Scientific Inc., Waltham, MA, USA). Then, 2 *μ*g of total RNA was used for stranded RNA sequencing library preparation using the KC-Digital™ Stranded mRNA Library Prep Kit for Illumina® following the manufacturer's instructions. The kit eliminates the duplication bias in the PCR and sequencing steps by using a unique molecular identifier (UMI) of 8 random bases to label the preamplified cDNA molecules. The library products were enriched, quantified, and sequenced on a Novaseq 6000 sequencer (Illumina, San Diego, MA, USA) in PE150 mode.

### 2.11. Bioinformatic Analysis

We analyzed the RNA-seq data using the *R* (4.0.5) software. Volcano plot was used to show differentially expressed genes in M2-MDEs and M0-MDE-treated meningioma cells. Based on the KEGG database, KEGG enrichment analysis was performed on genes significantly upregulated in tumor cells cocultured with M2 MDEs. GO enrichment analysis was performed using GSVA scores based on the MSigDB database. The relevant code is available from the author upon reasonable request.

### 2.12. Cell Viability Assay and EdU Staining

Cell viability was assessed using the CCK-8 method. Briefly, meningioma cells were plated in 96-well plates for 24–72 h, followed by the addition of 10 *μ*L of CCK-8 reagent (MCE) and incubation at 37°C for 2 h. The absorbance at 450 nm was measured using a microplate reader. For EdU staining, cells or 3D tumorspheres were incubated with 10 *μ*M EdU reagent (KeyGEN) for 2 h; they were subsequently trypsinized into cell suspensions and fixed using 4% paraformaldehyde. According to the instructions of the reagent's manufacturer, EdU-positive cells were fluorescently labeled with FITC, and the positive-cell ratio was determined using flow cytometry.

### 2.13. Animal Experiments

Five-week-old male athymic BALB/c nude mice (18–24 g) were provided by the HFK Bioscience (Beijing, China). The nude mice were housed in a 25°C room with a light/dark cycle every 12 hours. Food and water were freely available to the mice. For the construction of xenograft tumor models, IOMM-Lee (1 × 10^6^) cells were subcutaneously injected into the flank of mice. Starting on day 3, exosomes (10 *μ*g) were injected into mice via the tail vein every 3 days, and LY2109761 (50 mg/day) was administered by gavage. The tumor growth status was observed, and vernier calipers were used to measure the tumor's size for 18 days. After 18 days, the mice were sacrificed by cervical dislocation after administering anesthesia with isoflurane. The formula for calculating the volume of the xenograft tumor: volume = 0.5 × length × width^2^. All the animal experiments were approved by the Ethics Committee of West China Hospital (file No. 2021904A).

### 2.14. Statistical Analysis

The GraphPad Prism software (v8.0, USA) was used for statistical analysis. Student's *t*-test and one-way ANOVA were used to calculate the *p* values for differences between groups. When *p* < 0.05, differences were considered statistically significant.

## 3. Results

### 3.1. Macrophages Are Enriched in Meningioma Tissue, and Coculture with Meningioma Cells Promoted M2 Polarization of Macrophages

**Figure 1:**
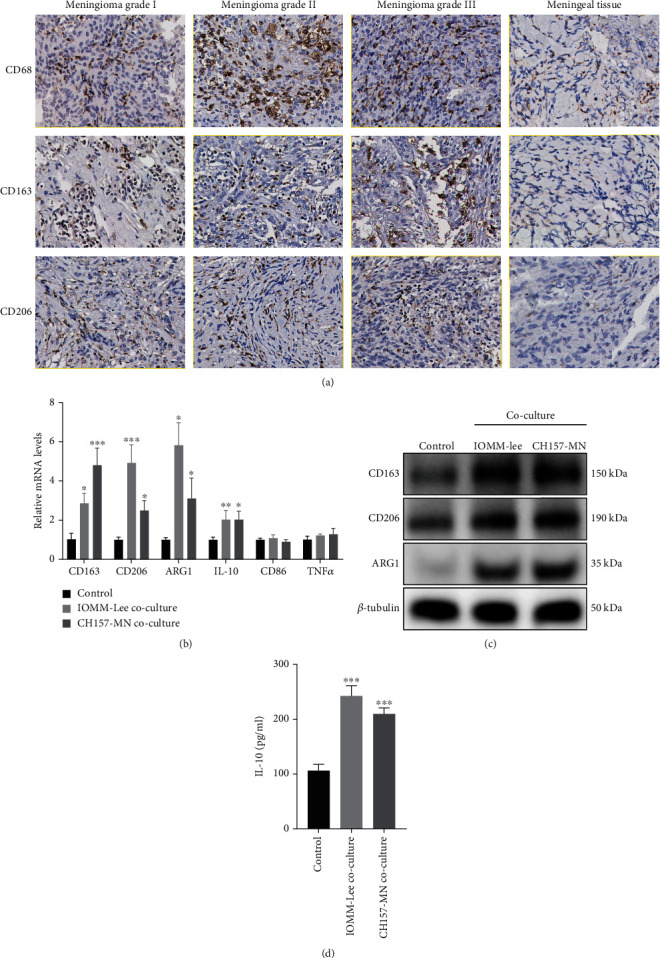
Macrophages are enriched in meningioma tissue, and coculture with meningioma cells promotes M2 polarization of macrophages. (a) Immunohistochemical staining was used to detect the distribution of macrophages and the expression levels of polarization markers in meningiomas and meningeal tissues. Scale bar 50 *μ*m. (b) Detection of mRNA expression levels of CD163, CD206, ARG1, IL-10, CD86, and TNF-*α* in macrophages by qPCR. (c) After coculture with tumor cells, the protein expression levels of CD163, CD206, and ARG1 in macrophages were detected by western blotting. (d) ELISA assay was used to detect the content of IL-10 secreted by macrophages in culture supernatant. ^∗^*p* < 0.05, ^∗∗^*p* < 0.01, ^∗∗∗^*p* < 0.001, vs. the control group.

To identify the infiltration and functional characteristics of macrophages in meningiomas and adjacent normal meningeal tissue, we performed IHC staining and analyzed the expression of CD68, CD163, and CD206. The IHC results show that macrophages were abundantly infiltrated in tumors relative to meningeal tissues (Figure 1(a)). Interestingly, CD163 and CD206 are ubiquitously expressed in these infiltrating macrophages, especially in high-grade meningiomas, suggesting that they may be induced to become TAMs with an M2 phenotype in the TME. Previous studies have provided evidence that the paracrine secretion of tumor in various malignant tissues is an inducer of the tumor-promoting phenotypic transformation of macrophages [30, 31]. In vitro, THP-1 was induced to differentiate into M0 macrophages by coculture with 100 ng/mL of phorbol 12-myristate 13-acetate (PMA) for 24 hours. In order to explore the paracrine effect of meningioma cells on macrophage polarization, we constructed a noncontact tumor–macrophage coculture system by using Transwell chambers. We assessed the expression levels of polarization markers in macrophages after 48 hours of coculture with meningioma cell lines. As shown, macrophages cocultured with IOMM-Lee and CH157-MN cells had higher mRNA levels of CD163, CD206, IL-10, and ARG1 as detected by qPCR assay (Figure 1(b)). Similar results were detected in western blot (Figure 1(c)). At the same time, we detected greater IL-10 secretion by macrophages cocultured with meningioma cells by ELISA (Figure 1(d)). These results indicate that macrophages are enriched in meningioma tissue and exhibit M2-prone polarization during coculture with tumor cells.

### 3.2. Characterization of Macrophage-Derived Exosomes and Internalization

THP-1 was induced to differentiate into M0 macrophages in the presence of 100 ng/mL PMA for 24 hours and subsequently cocultured with IL-4 and IL-13 to induce M2-phenotype polarization. Macrophage-derived exosomes (MDEs) were collected by ultracentrifugation. The detection results of transmission electron microscopy (TEM) and the nanoparticle tracking analyzer indicated that MDEs exhibited morphological characteristics typical of exosomes (Figure S1A, B). Western blotting showed that CD81, TSG101, and CD9 were highly enriched in M2-MDEs and M0-MDEs, all of which are positive markers of exosomes (Figure S1C). And calnexin, an intracellular calcium-binding protein considered to be a negative marker for exosomes, was absent or underexpressed in isolated MDEs. To examine whether meningioma cells could take up MDEs, we incubated them with PKH67-labeled MDEs. Green fluorescence was observed outside the DAPI-labeled nuclei, which suggested that M0-MDEs and M2-MDEs could be phagocytosed by tumor cells (Figure S1D).

### 3.3. M2-Macrophage-Derived Exosomes Promote Meningioma Cell Proliferation and Inhibit Apoptosis

**Figure 2:**
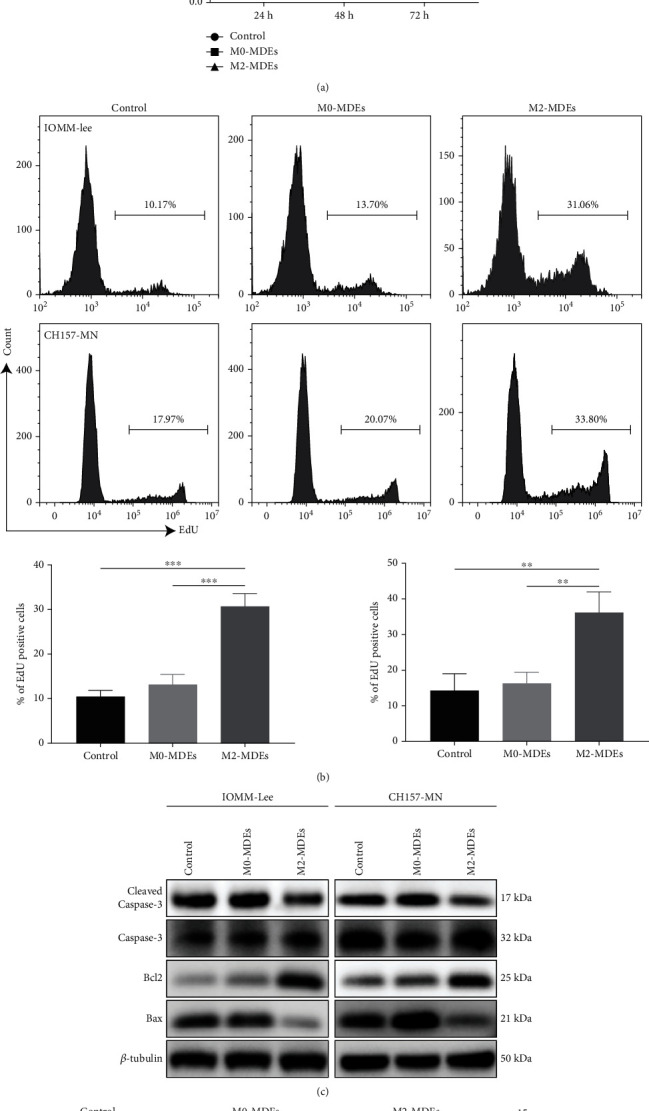
M2-macrophage-derived exosomes promote meningioma cell proliferation and inhibit apoptosis. (a) Detection of cell viability during 24–72 h proliferation in IOMM-Lee and CH157-MN cells using CCK-8 assay. (b) EdU staining and flow cytometry to detect the fraction of proliferating cells in IOMM-Lee and CH157-MN cells. (c) Western blotting was used to detect the protein expression levels of caspase-3, cleaved-caspase-3, Bcl2, and Bax. (d) FITC-conjugated Annexin V kit was used for labeling apoptotic cells in IOMM-Lee and CH157-MN cells, and distribution was detected by flow cytometry. ^∗^*p* < 0.05, ^∗∗^*p* < 0.01, ^∗∗∗^*p* < 0.001, vs. the control and M0-MDE group.

Previous studies suggested an important role for TAM-derived exosomes in tumor proliferation and antiapoptotic processes. To further investigate the role of M2-MDEs in meningioma progression, we coincubated meningioma cells with M0-MDEs and M2-MDEs separately for 48 h and then detected the proliferative activity and apoptosis levels of the tumor cells. As shown in Figure 2(a), the CCK-8 results indicate that, compared with the control and M0-MDEs group, meningioma cells cocultured with M2-MDEs exhibited higher proliferative activity, while M0-MDEs failed to promote tumor cell proliferative activity. Similarly, a higher proportion of EdU^+^ tumor cells was identified in the M2-MDE coculture group by flow cytometry, suggesting that M2-MDEs, but not M0-MDEs, can promote tumor cell mitosis (Figure 2(b)). The expression of apoptosis-related proteins in meningioma cells was further examined by western blotting. As shown in Figure 2(c), the expression of the antiapoptotic protein Bcl2 was significantly increased in tumor cells after M2-MDE treatment, while the expression of the proapoptotic protein Bax was downregulated. The expression of the apoptotic marker cleaved-caspase-3 was downregulated in meningioma cells after M2-MDE treatment but did not change significantly in the M0-MDE group. Furthermore, we detected the fraction of Annexin V/PI-positive cells by flow cytometry to characterize early and late apoptotic cell death (Figure 2(d)). The results showed that the proportion of apoptotic cells was significantly reduced in both IOMM-Lee and CH157-MN after 48 hours of treatment with M2-MDEs. Overall, according to our results, coculture with M2-MDEs can specifically promote the proliferative viability and reduce the level of apoptosis of meningioma cells.

### 3.4. M2-MDEs Promote Metastasis of Meningioma Cells

**Figure 3:**
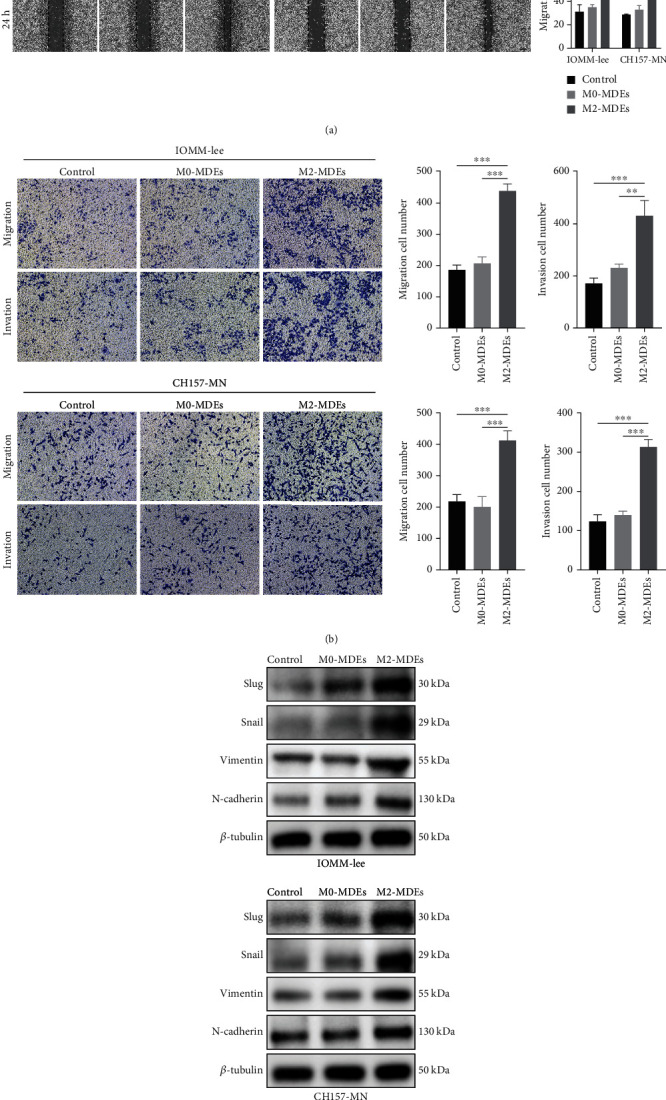
M2-MDEs promote the migration and invasion of meningioma cells. (a) The wound healing assay was used to examine the mobility of IOMM-Lee and CH157-MN cells. Scale bars 200 *μ*m. (b) The migration and invasion ability of IOMM-Lee and CH157-MN cells was assessed using Transwell chambers, and the number of cells passing through the upper chamber membrane was counted. Scale bars 100 *μ*m. (c) Protein expression levels of slug, snail, vimentin, and N-cadherin were detected by western blotting. ^∗^*p* < 0.05, ^∗∗^*p* < 0.01, ^∗∗∗^*p* < 0.001, vs. the control and M0-MDE group.

Next, we tried to delve into the effect of M2-MDEs on the migration and invasion ability of meningioma cells. The wound healing assay was used to quantify meningioma cell mobility. Meningioma cells cocultured with M2-MDEs exhibited increased mobility compared to the control and M0-MDE groups (Figure 3(a)). Transwell assays were subsequently performed, and M2-MDEs induced a significant increase in the number of tumor cells migrating across the membrane to the lower chamber (Figure 3(b)). In addition, we preincubated Matrigel on the membrane of the upper Transwell chamber and then examined the tumor cell invasiveness. The results indicate that M2-MDE-treated meningioma cells exhibited a stronger invasive ability (Figure 3(b)). It is well known that EMT is highly correlated with tumor invasion and metastasis, and transformation to a mesenchymal-like phenotype confers enhanced motility of tumor cells. Therefore, we suspected that M2-MDEs might have an impact on the EMT process and related signaling pathways in meningioma cells. To assess whether coculture with M2-MDEs induces EMT processes in meningioma cells, the expressions of canonical EMT markers were detected. As shown in Figure 3(c), the protein expression levels of mesenchymal markers slug, snail, vimentin, and N-cadherin in meningioma cells were significantly increased after treatment with M2-MDEs for 48 h, relative to the control and M0-MDE groups. In short, our results suggest that coculture with M2-MDEs increases the migratory and invasive abilities of meningioma cells.

### 3.5. TGF-*β* Pathway Is Activated in M2-MDE-Treated Meningioma Cells

**Figure 4:**
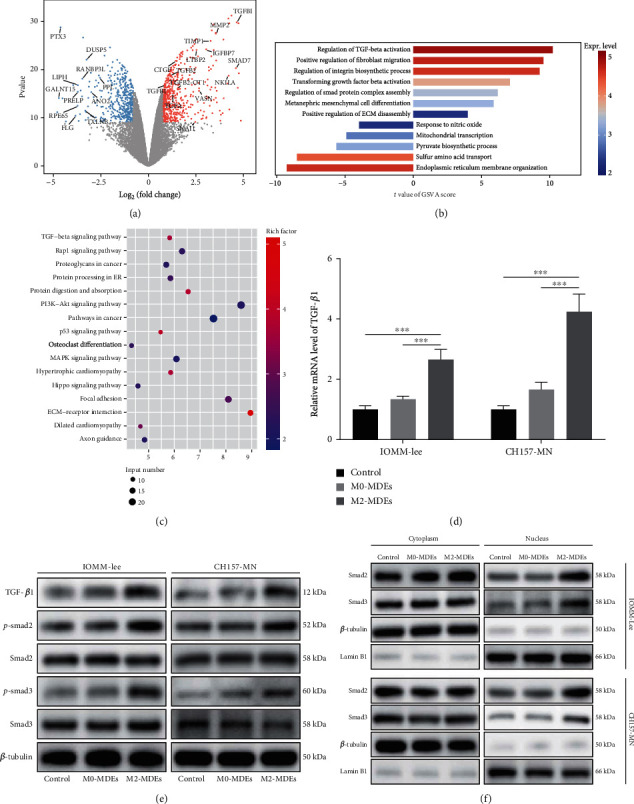
The TGF-*β* pathway is activated in M2-MDE-treated meningioma cells. (a) Volcano plot showing differentially expressed genes in IOMM-Lee cells cocultured with M2-MDEs relative to M0-MDEs. (b) GO enrichment analysis was used to reveal the functional characteristics of IOMM-Lee after coculture with MDEs according to the MSigDB database. (c) KEGG pathway analysis of IOMM-Lee after coculture with MDEs based on differentially expressed genes0. (d) qPCR was used to detect the mRNA expression level of TGF-*β*1 in meningioma cells. (e) Western blotting was used to detect the protein expression levels of TGF-*β*1, Smad2, p-Smad2, Smad3, and p-Smad3 in meningioma cells. (f) Western blotting for the expressions of Smad2 and Smad3 in the cytoplasm and nucleus of meningioma, respectively. *β*-Tubulin and lamin B1 serve as house-keeping genes for cytoplasmic and nuclear proteins, respectively. ^∗^*p* < 0.05, ^∗∗^*p* < 0.01, ^∗∗∗^*p* < 0.001, vs. the control and M0-MDE group.

To explore the molecular mechanism by which M2-MDE regulates the proliferative activity and invasive ability of meningioma cells, we performed RNA-seq to explore the transcriptome differences in meningioma cells cocultured with M0-MDEs and M2-MDEs. Differential gene analysis and pathway enrichment analysis revealed potential differences in biological functions in the tumor cells (Figures 4(a)–4(c)). We noticed that genes such as TGFBI, SNAI1, MMP2, TGFB1, TGFB2, IGFBP7, and LTBP2 were upregulated by the treatment of M2-MDEs in meningioma cells and may contribute to tumor invasion and proliferation behavior. In particular, GO enrichment analysis suggested that multiple TGF-*β* signaling-related pathways including “Regulation of TGF-beta activation” and “Regulation of smad protein complex assembly” were activated in meningioma cells cocultured with M2-MDEs, which was similar to the trend of the results of KEGG analysis. Previous studies have not reported the functional characterization for the TGF-*β* signaling in macrophage-tumor communication in the meningioma microenvironment. Therefore, we hypothesized that the TGF-*β* pathway might be closely related to the protumor biological effects induced by M2-MDEs, and we validated the RNA-seq conclusions at the mRNA level. The results of qPCR suggested that the expression of TGF-*β*1 was upregulated by the treatment of M2-MDEs, which supported the above results (Figure 4(d)). Furthermore, we analyzed the protein expression levels of key molecules of the TGF-*β* pathway in meningioma cells by western blotting (Figure 4(e)). The results showed that coculture with M2-MDEs increased the phosphorylation level of Smad2/3 in meningioma cells. As classical downstream transcription factors of the TGF-*β* pathway, translocation of phosphorylated Smad2/3 into the nucleus is a hallmark of TGF-*β* pathway activation. The western blotting of cytoplasmic and nuclear proteins revealed increased nuclear translocation of Smad2 and Smad3 in meningioma cells cocultured with M2-MDEs (Figure 4(f)). In short, our study suggests that the activation of the TGF-*β* pathway may be a key biological event for M2-MDEs' promotion of tumor progression.

### 3.6. Inhibition of TGF-*β* Pathway Reverses the Tumor-Promoting Biological Effects Mediated by M2-MDEs

**Figure 5:**
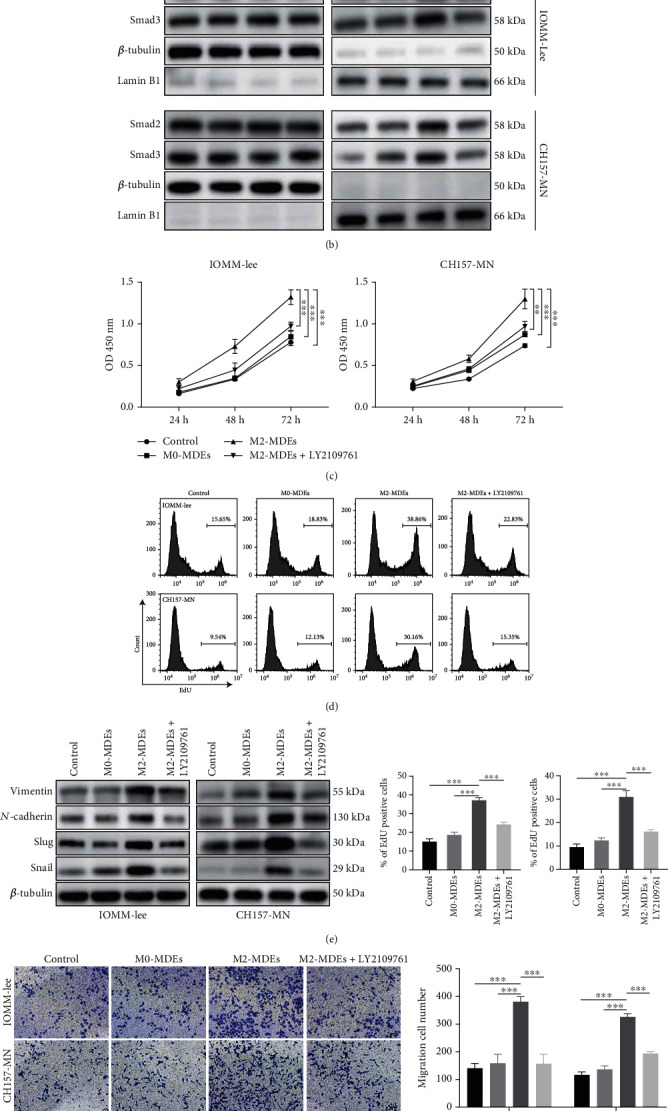
Inhibition of the TGF-*β* pathway reverses the tumor-promoting biological effects mediated by M2-MDEs. (a) Western blot detection of protein expressions of TGF-*β*1, p-Smad3, Smad3, p-Smad2, and Smad2. (b) The expressions of Smad2 and Smad3 in nuclear and cytoplasmic protein fractions, respectively, were detected by western blotting. *β*-Tubulin and lamin B1 serve as house-keeping genes for cytoplasmic and nuclear proteins, respectively. (c) Detection of cell viability of IOMM-Lee and CH157-MN cells by CCK-8 assay. (d) EdU staining was used to label tumor cells in the proliferating state, and the EdU+ cell ratio was detected by flow cytometry. (e) Western blotting for the expressions of EMT-related genes. (f) Transwell chambers were used to detect tumor cell migration ability. (g) Transwell chambers preincubated with Matrigel were used to assess tumor cell invasiveness. ^∗^*p* < 0.05, ^∗∗^*p* < 0.01, ^∗∗∗^*p* < 0.001.

The above results raised the question of whether the TGF-*β* signaling pathway was responsible for the tumor-promoting effects of M2-MDEs. Thus, LY2109761, a classical selective TGF-*β*-receptor type-I inhibitor, was used to block TGF-*β* signaling. Western blotting demonstrated that LY2109761 not only effectively reduced the phosphorylation of Smad2/3 but also prevented their nuclear translocation, indicating that the upregulation of the TGF-*β* signaling in tumor cells induced by M2-MDE coculture was successfully inhibited (Figures 5(a) and 5(b)). At the same time, we found that inhibition of TGF-*β* pathway partially reversed the promoting effect of M2-MDEs on meningioma cell proliferation detected by CCK8 assay and EdU staining (Figures 5(c) and 5(d)). Furthermore, we observed similar results for meningioma cell migration and invasion. Western blotting revealed that the epithelial–mesenchymal transition of meningioma cells is reversed by combination treatment with LY2109761 (Figure 5(e)). Similarly, LY2109761 inhibited the migratory and invasive abilities of meningioma cells as detected by Transwell assays with or without Matrigel (Figures 5(f) and 5(g)). In short, our in vitro studies confirmed that the effects of M2-MDEs on meningioma cells, including cell viability, migration, and invasion, could be reversed by blocking the TGF-*β* signaling pathway.

### 3.7. M2-MDEs Promote Tumorigenesis of Meningioma Cells In Vitro and In Vivo

**Figure 6:**
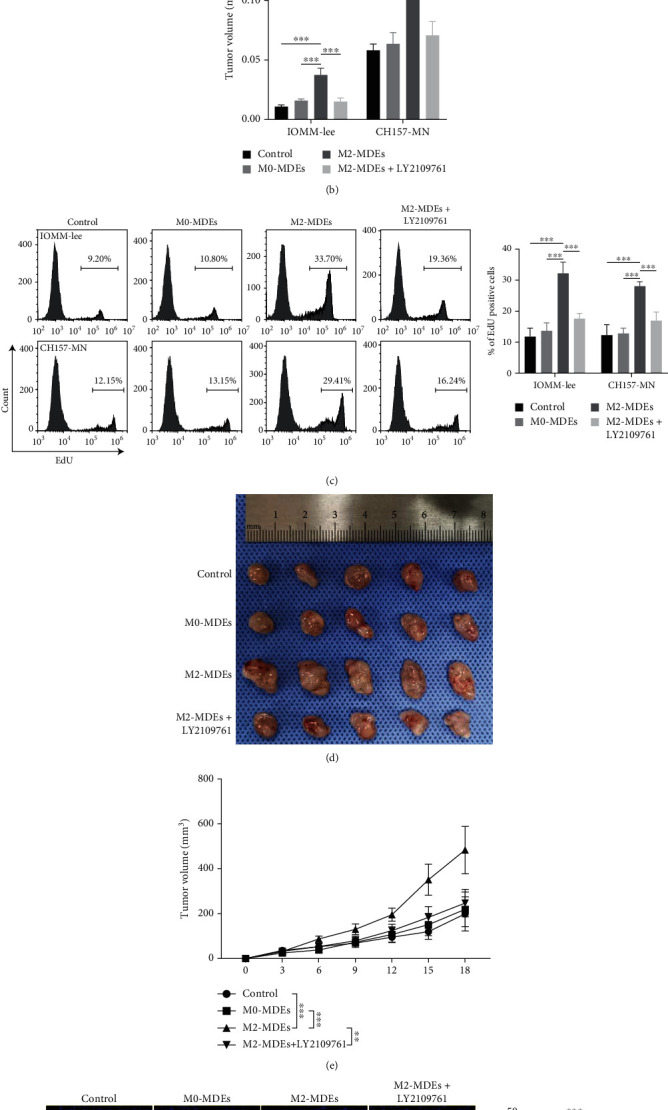
M2-MDEs promote tumorigenesis of meningioma cells in vitro and in vivo. (a) 3D tumorspheres formed in vitro by meningioma cells cocultured with MDEs. Scale bar 200 *μ*m. (b) Measurement and statistical analysis of the volume of 3D tumorspheres. (c) Labeling of proliferating meningioma cells in 3D tumorspheres with EdU staining and detection of EdU^+^ cell ratio by flow cytometry. (d, e) Dissection of subcutaneous xenografts and measurement of tumor volume. (f) Proliferating cells were identified by immunofluorescence using antibody against Ki-67. Tumor proliferative activity was evaluated by calculating the proportion of Ki-67^+^ cells. ^∗^*p* < 0.05, ^∗∗^*p* < 0.01, ^∗∗∗^*p* < 0.001.

To comprehensively elucidate the potential role of M2-MDEs on meningioma tumorigenesis and progression, we constructed a 3D tumorsphere model and cocultured it with MDEs. As shown, 3D tumorspheres cocultured with M2-MDEs had larger volumes (Figures 6(a) and 6(b)). To elucidate the proliferative activity of meningioma tumorspheres, the tumorspheres were enzymatically dissociated into single-cell suspensions after incubation with EdU reagent on the last day of in vitro observation. The EdU^+^ cell fraction detected by flow cytometry indicated that M2-MDE treatment could significantly promote the proliferative activity of meningioma cells that constitute 3D tumorspheres (Figure 6(c)). Treatment with LY2109761 effectively inhibited the promoting effects of M2-MDEs on the development of 3D tumorspheres in meningiomas. The meningioma cell line IOMM-Lee was injected subcutaneously into nude mice to form xenograft tumors. To assess the critical role of M2-MDEs in meningioma tumorigenesis in vivo, M2-MDEs and M0-MDEs were injected into tumor-bearing mice via tail vein injection. LY2109761 was administered by intragastric gavage. The results show that M2-MDEs significantly promoted meningioma development relative to M0-MDEs over an observation period lasting 3 weeks (Figures 6(d) and 6(e)). Treatment with LY2109761 effectively reduced the sizes of the xenograft tumors. Immunofluorescence staining for Ki-67 revealed higher cell viability in M2-MDE-treated xenograft tumors, which was reversed in the LY2109761-treated group (Figure 6(f)). In short, our study demonstrates that M2-MDEs play a promoting role in meningioma tumorigenesis, which can be partially reversed by blocking TGF-*β* signaling.

## 4. Discussion

Patients with malignant meningiomas tend to have poor prognoses and high mortality due to the uncontrollable proliferative activity and invasive ability of tumor cells. Despite improvements in the treatment of meningiomas over the past decade, the role of the TME in meningioma development remains unclear. Previous studies have suggested that a variety of stromal cells, including TAMs, interact with tumor cells in the TME, which contributes to tumor progression [14, 15, 17]. However, the molecular mechanisms underlying the integral role of infiltrating TAMs in the development of malignant meningiomas have not been fully studied. In recent years, the immunological landscape in meningiomas has been reported in the literature, and tumor-associated macrophages are the dominant immune population in meningioma tissues, suggesting their criticality in tumor progression. In our study, the IHC analysis revealed that TAMs were enriched in meningioma tissue relative to meningeal and brain tissue. Infiltrating TAMs in meningiomas exhibit M2-biased polarization, which has been reported to assist tumor progression. Based on these results, we speculate that unique cell-to-cell communication in the TME might induce a transition in the functional state of the TAMs. It has been reported that the paracrine secretion of various malignant cells, including lung cancer and colorectal cancer cells, can induce the differentiation of TAMs to an anti-inflammatory phenotype. Our results from noncontact coculture experiments show that the paracrine signaling of meningioma cells induces the M2 polarization of TAMs.

Accumulating evidence supports a unique role of exosomes in intercellular communication in the TME as widely distributed transport mediators in the stroma [32, 33]. For example, Yang and colleagues proposed that exosomes from M2 macrophages promoted angiogenesis and tumor growth in pancreatic cancer in an E2F2-dependent manner [12]. The WHO classification of meningiomas is based on the number of proliferating cells. The most prominent features of grade III meningiomas are abnormally increased cell viability and mitotic rates, which pose challenges for the treatment of malignant meningiomas. In this study, we found that M2-MDEs could significantly promote the cell viability and inhibit the apoptosis of meningioma cells, which may explain the abnormal cell proliferation in malignant meningioma. Targeting infiltrating macrophages and M2-MDEs in tumor tissues may provide ideas for clinical therapeutic strategies to curb the proliferation of malignant meningiomas. Furthermore, increasing evidence suggests that M2 TAM-derived exosomes promote malignant tumor invasion and metastasis. Lan et al. demonstrated that M2-MDEs promoted colon cancer cell invasiveness and metastasis by delivering miR-21-5p and miR-155-5p [34]. High-grade meningiomas often exhibit creeping growth due to their enhanced invasive capacity, which leads to higher surgical risk and poorer prognosis [35, 36]. Interestingly, our study showed that coculture with M2-MDEs conferred enhanced migratory and invasive abilities to meningioma cells. Furthermore, M2-MDEs facilitated the upregulation of vimentin, N-cadherin, slug, and snail in meningioma, suggesting that M2-MDEs contribute to the mesenchymal transition of meningioma cells.

Multiple signaling pathways have been shown to be involved in M2-MDE-mediated biological effects. Here, we performed RNA-seq to analyze the differential transcriptomes of tumor cells cocultured with M2-MDEs and M0-MDEs individually. We identified the TGF-*β* signaling pathway as a potential mechanism responsible for the regulation of tumor development and invasion by M2-MDEs. Previous studies have also shown that the TGF-*β* pathway is involved in the regulation of tumor EMT and mobility. Similar to previous studies, our study shows that M2-MDEs can effectively induce the phosphorylation of Smad2/3 and promote their nuclear accumulation relative to M0-MDEs. Correspondingly, blocking the TGF-*β* pathway with selective small-molecule inhibitors significantly reversed the increase in meningioma cell viability and invasive capacity induced by M2-MDEs. Therefore, targeting the M2-MDE-mediated activation of the TGF-*β* pathway in tumor cells may be a potential therapeutic strategy for meningiomas.

Subsequently, we further explored the effects of M2-MDEs on tumorigenesis and tumor development by constructing 3D tumorspheres and xenograft tumor models. Compared with that of 2D culture, the growth environment of 3D tumorspheres allows meningioma cells to exhibit characteristics closer to those in the natural conditions in vivo [37]. Multiple studies suggest that M2-macrophage infiltration contributes to tumor development. However, depending on the cell type and environmental characteristics, the TGF-*β* signaling pathway plays a binary role in tumorigenesis. In our study, coculture with M2-MDEs effectively promoted tumor growth and cell viability in 3D tumorspheres and xenografts. Consistent with 2D cultures, these biological effects were reversed by treatment with LY2109761.

It is worth noting that our work also has certain limitations. First, our study did not focus on the source of infiltrating macrophages in the meningioma microenvironment, which may contribute to understanding the mechanisms of meningioma tumorigenesis. Second, the transformation of the functional state of infiltrating macrophages in meningioma tissue and its molecular mechanisms remains to be further explored. Based on the results of this study, characterizing the contents of infiltrating macrophage-derived exosomes to explore the molecular mechanisms of their protumor biological effects in meningiomas will be a further research strategy.

In short, our study confirmed that M2-MDEs promote the proliferation, migration, and invasion of meningioma cells by activating the TGF-*β* signaling pathway. Therefore, targeting TAM-derived exosomes is expected to be a promising treatment modality for meningiomas. Based on the underlying intercellular communication network in the TME, this article provides a novel idea for drug treatment strategies for meningioma.

## Data Availability

All data supporting this study is available from the corresponding author on reasonable request.

## Supplementary Materials

Supplementary Materials Figure S1: characterization of macrophage-derived exosomes and internalization. (a) Morphology of exosomes observed by transmission electron microscopy. Scale bars 200 nm. (b) The size distribution of MDEs determined by nanoparticle tracking analysis. (c) Expression levels of exosomal markers CD81, TSG101, CD9, and calnexin in MDEs were detected by western blotting. (d) After coculturing PKH67-labeled MDEs with meningioma cell lines IOMM-Lee and CH157-MN for 2 hours, green fluorescence was distributed inside the tumor cells. Table S1: list of primary antibodies. Table S2: primer sequences for quantitative real-time PCR reactions.
