# Supplementary figures and images for: M2-Macrophage-Derived Exosomes Promote Meningioma Progression through TGF-β Signaling Pathway
Source: J Immunol Res. 2022 May 20;2022:8326591. doi: 10.1155/2022/8326591 (PMC9146444; doi:10.1155/2022/8326591)

A

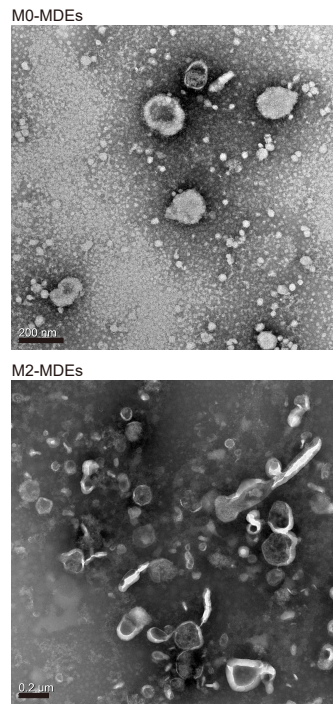

B

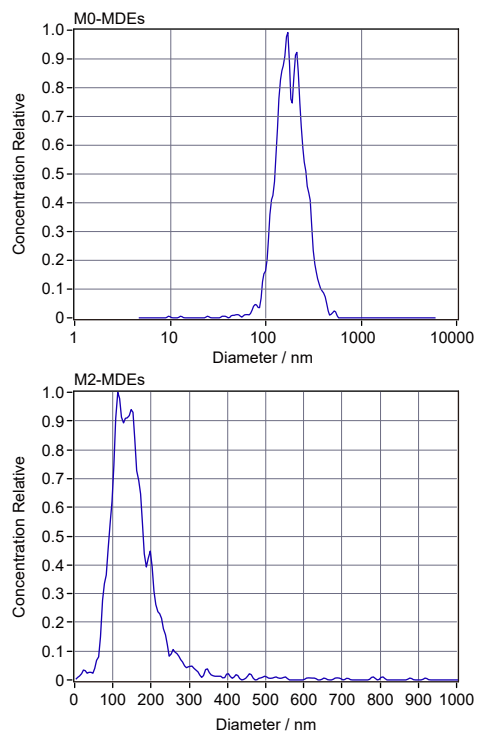

C

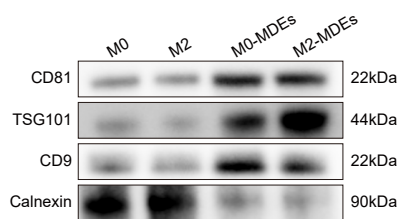

D

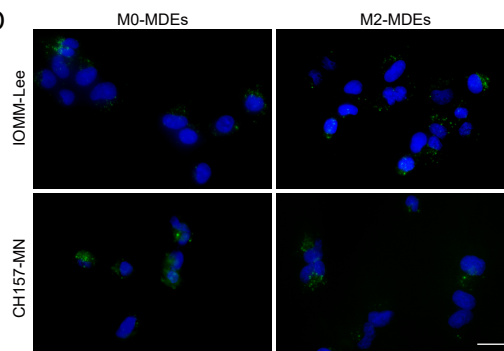

Supplement: Supplementary Materials — Figure S1: characterization of macrophage-derived exosomes and internalization. (a) Morphology of exosomes observed by transmission electron microscopy. Scale bars 200 nm. (b) The size distribution of MDEs determined by nanoparticle tracking analysis. (c) Expression levels of exosomal markers CD81, TSG101, CD9, and calnexin in MDEs were detected by western blotting. (d) After coculturing PKH67-labeled MDEs with meningioma cell lines IOMM-Lee and CH157-MN for 2 hours, green fluorescence was distributed inside the tumor cells. Table S1: list of primary antibodies. Table S2: primer sequences for quantitative real-time PCR reactions. [file 8326591.f1.zip › 8326591.f1/FigureS1 (1).pdf]
